# Genetic Diversity in Invasive Populations of *Lupinus polyphyllus* Lindl. and *Heracleum sosnowskyi* Manden.

**DOI:** 10.3390/biology10111094

**Published:** 2021-10-25

**Authors:** Ekaterina Sergeevna Osipova, Anna Yurievna Stepanova, Dmitry Viktorovich Tereshonok, Evgeny Aleksandrovich Gladkov, Olga Nikolaevna Vysotskaya

**Affiliations:** K.A.Timiryazev Institute of Plant Physiology, Russian Academy of Sciences, 127276 Moscow, Russia; step_ann@mail.ru (A.Y.S.); diman_ter_vi@mail.ru (D.V.T.); gladkovu@mail.ru (E.A.G.); cryo_ippras@mail.ru (O.N.V.)

**Keywords:** lupin, hogweed, *Lupinus polyphyllus* Lindl., *Heracleum sosnowskyi* Manden., invasive population, native population, feral population, RAPD, ISSR, REMAP, genetic diversity, phylogenetic tree

## Abstract

**Simple Summary:**

Aggressive-invasive species often interact with native ones, thus considerably changing the biological communities, with ecological, economic, and even social effects. It is a challenge to evaluate the direction and the rate of microevolution in native and introduced populations. One of the ways to do this is to estimate the genetic diversity. An introduction often imposes a reduction in population size (genetic drift, bottleneck, founder effect), which has the potential to reduce genetic diversity. However, after a lag, the genetic diversity can be restored due to repeated invasions (multiply introductions), hybridization between individuals from two different subspecies or species in the invaded ranges, as well as during rapid genetic changes under selection pressures in the novel environment. The purpose of this study was to determine the level of genetic diversity in successful invasive species *Lupinus polyphyllus* Lindl. and *Heracleum sosnowskyi* Manden. from Russia and Ukraine, and whether it may be associated with the strategy of their further expansion.

**Abstract:**

In our study, two aggressive-invasive species, *Lupinus polyphyllus* Lindl. and *Heracleum sosnowskyi* Manden. from Russia and Ukraine, were investigated. The success in naturalization of both species is associated with human activities, since they have been used in agriculture and floriculture and both have qualities such as environmental tolerance, high fertility and phenotypic plasticity. The purpose of this study was to determine the level of genetic diversity of both species. For *Heracleum sosnowskyi* Manden., genetic diversity was compared in invasive and native populations. For *Lupinus polyphyllus* Lindl., the genetic diversity was compared in variety, feral and invasive populations. A genetic diversity was formulated using RAPD, ISSR and REMAP. For *Heracleum sosnowskyi* Manden., the average genetic diversity within the invasive population was similar (0.432), but slightly less (0.502) than within the native Caucasian population. This may suggest the successful naturalization of invaders and almost complete reconstruction of their genetic diversity. For *Lupinus polyphyllus* Lindl., the genetic diversity for the invasive population was the highest, with an average of 0.294, while for variety, it was the lowest, with an average of 0.194. The feral population had an intermediate place with an average of 0.248, which could suggest an increase of diversity in the process of naturalization.

## 1. Introduction

Invasive-alien species (IAS) can be considered the second most important threat to biodiversity, after habitat destruction [1]. When introduced into the ecosystem, IAS directly or indirectly affect human health [2]. Warnings from the scientific community for this serious environmental problem have led to the emergence of special international agreements and programs operating under the auspices of several trustworthy organisations. The issue of IAS is addressed in Europe by EU Regulation 1143/2014 of the European Parliament and Council. To support the decision of the EU IAS Regulation, it is important to organize a participatory approach involving scientists and practitioners, draft realistic management strategies for eradication and spread limitation of IAS [3]. Sometimes amendments to legislation may even be necessary [4]. The list of Invasive Alien Species of Union Concern is regularly updated [5]. The preliminary list of invasive plants in Russia includes 730 species [6]. In our work, two aggressive-invasive species, *Lupinus polyphyllus* Lindl. and *Heracleum sosnowskyi* Manden. From Russia and Ukraine, were studied.

*L*. *polyphyllus* is an herbaceous biennial or short-lived perennial of the Fabaceae with a height of 0.8–1.5 m. The flowers (up to 80 pieces) are blue, or less often pink or white, and are grouped in a terminal erect inflorescence. The natural habitat of *L*. *polyphyllus* is western North America—Canada (British Columbia) and the United States (Alaska, western Oregon and Washington, northern California). This species grows on riverbanks, meadows, roadsides, and the other disturbed habitats [7]. In 1826, the famous Scottish ‘plant hunter’ David Douglas brought *L*. *polyphyllus* to England, and soon lupins were cultivated in Europe as garden plants. As early as the 1840s, botanical gardens offered a variety of colour forms for this species. The distribution of lupins in Europe had increased by the beginning of the 20th century, as they were grown for soil amelioration and animal feed [8]. Soil amelioration occurred due to the symbiosis between *L*. *polyphyllus* and *Bradyrhyzobium* spp., which form nitrogen-fixing root nodules [9]. In Germany, lupins were grown especially on poor, acidic soils in mountainous regions. In Northern Europe (Norway, Baltic countries), lupins were used to stabilise the soil substrate [10], while in Belarus, Poland, and Russia, they were cultivated for green biomass and soil amelioration [11,12]. In Lithuania and Belarus, lupins were also used as a precautionary measure against the spread of forest fires [13]. In Russia, *L*. *polyphyllus* became widespread, to the extent that it became listed in the “Central Russia Floral Black Book” [14]. 

*H*. *sosnowskyi*, or hogweed, is a perennial monocarpic herbaceous plant of the Umbelliferae with a height of 3–4 m. The inflorescence is a large complex umbel (up to 50–80 cm in diameter) consisting of 30–75 rays. The flowers are white or, less often, pink, and each inflorescence has from 30 to 150 flowers. Thus, there can be more than 80,000 flowers on one plant. The natural range of hogweed is east of the Greater Caucasus, the east and southeast of Transcaucasia, and the northeast of Turkey. It grows in subalpine meadows, glades, and edges of beech and fir-beech forests in the middle and upper forest belt [15]. The plant was first described in 1944 in Georgia by botanist I. P. Mandenova. It is named after the famous explorer of flora of the Caucasus D. I. Sosnovsky [16]. Since 1947, *H*. *sosnowskyi* has been cultivated throughout the former USSR as a highly productive and stable silage plant. The active cultivation of *H*. *sosnowskyi* was promoted because of its extraordinary productivity (its green biomass is 3–4 times higher than all previously known silage crops) and high protein (10–20%), sugar (10–31%), vitamins, and mineral elements [17]. Selective breeding developed varieties with low furanocoumarins content (for example, the variety ‘Severyanin’ was created in the Komi Republic by Mishurov V.M.). Furanocoumarins are photosensitising compounds present in the cellular fluid of hogweed; they possess various physiological activities, including the increase of skin sensitivity to ultraviolet rays. Moreover, it was found that even in the absence of photoactivation, *H*. *sosnowskyi* cellular fluid has several pathogenic properties; it inhibits cell growth and leads to cell death, oppresses mitosis, induces chromosomal mutations and causes apoptosis [18]. The milk and meat of cows that have been fed for a long time on *H. sosnowskyi* acquire an unpleasant bitter taste, and then the phytoestrogens that are part of the plant induce infertility in animals. Consequently, when all these unpleasant side-effects became clear, the breeding of *H. sosnowskyi* was stopped. By the early 1980s, using hogweed for silage was mostly abandoned; however, the plant turned out to be very tenacious, and feral populations developed quickly. By 1990–2000, hogweed became a serious problem in many ranges of the European part of Russia. The spread of *H*. *sosnowskyi* was facilitated by the abandonment of once cultivated lands that occurred with the decline of agriculture following the collapse of the USSR. The consequence then was that hogweed began to spread especially actively. Thus, *H. sosnowskyi* is called “an indicator of mismanagement” [19]. *H*. *sosnowskyi* increased in its growth area by 10% annually, with its invaded ranges including the countries of the Baltic region (Denmark, Poland, Estonia, Lithuania, Latvia), Belarus, Ukraine, and Russia. In Russia, hogweed has spread widely in the European part of Russia, penetrating the Urals, the south of Western Siberia, and the Far East. It grows on the outskirts of roads, fields and forest belts, wastelands, forests, and floodplain meadows [20]. An active search for methods to control *H*. *sosnowskyi* began in the 2000s, with one of the first projects being the Giant Alien Project (2002–2005), dedicated to three giant hogweeds (*H*. *mantegazzianum* Sommier & Levier, *H*. *sosnowsky*, and *H*. *persicum* Desf. ex Fisch.). More than 40 scientists from seven countries participated, proposing, methods to combat all three species [20,21]. 

Upon introduction of a new species in an invaded range, the level of genetic diversity within populations is often reduced [22]. However, after a lag the genetic diversity can be restored [23,24,25]. Therefore, by understanding the level of genetic diversity it should be possible to predict the further spread of invasion. One of the most accessible, fast, and inexpensive techniques for detecting genetic diversity at the DNA level is a variant of PCR, multiple arbitrary amplicon profiling (MAAP), which includes Random Amplified Polymorphic DNA (RAPD) [26], Inter Simple Sequence Repeats (ISSR) [27], Retrotransposon-Microsatellite Amplified Polymorphisms (REMAP) [28]. Thus, our objectives were to estimate the genetic diversity in populations of *L. polyphyllus* and *H. sosnowskyi* by means of RAPD, ISSR, and REMAP. The genetic diversity of invasive populations of *H. sosnowskyi* was compared with its native population. The genetic diversity of invasive population of *L. polyphyllus* was compared with its variety and feral population, as examples at the beginning of the invasive process. 

## 2. Materials and Methods

### 2.1. Plant Material

Plant material used in this study included 84 L. polyphyllus and 99 *H. sosnowskyi* samples (Table 1 and Table 2) from different Russian and Ukrainian regions (Supplementary Figures S1 and S2). Leaf material was collected in the summers of 2019 and 2020 from individual plants at a minimum of 10 m apart. Leaves were desiccated in silica gel until completely dried. ‘Minaret’ variety samples were grown in the greenhouse of K.A.Timiryazev Institute of Plant Physiology, Moscow. Feral population was obtained from a variety that had been running wild for about 5 years in the Main Botanical Garden. Feral population consisted of naturalized plants or established plants. Pyshek characterized such plants as self-replacing populations sustained for at least 10 years without direct intervention by people capable of independent growth [29]. They are already not variety, but are not yet invasive.

### 2.2. DNA Isolation

DNA was isolated according to Dellaporta et al. [30] with the procedure scaled down for 50 mg dry leaves. As a negative control, C1-, water was added to the lysis buffer instead of plant material.

### 2.3. DNA Amplification

Twenty-five microlitres of the reaction mixture contained 0.25 µM primer (Lytech, Moscow, Russia), 0.2 mM each dNTP, 2U of Taq DNA polymerase, standard 10× PCR buffer (700 mM Tris-Cl pH 8.6, 166 mM (NH_4_)_2_SO_4_ and 25 mM MgCl_2_) (Silex, Moscow, Russia) and 30 ng of DNA. The reaction mixture was overlayed by 30 µL of mineral oil. Following optimization, the amplification conditions were: denaturation at 94 °C for 2 min, 5 cycles as follows: denaturation at 94 °C for 20 s, annealing at t °C for 10 c, elongation at 72 °C for 10 c; 35 cycles as follows: denaturation at 94 °C for 5 c, annealing at t °C for 5 c, elongation at 72 °C for 5 c; 1 cycle as follows: elongation for 2 min at 72 °C; t °C was 37 °C for RAPD primers, 37–55 °C for ISSR primers and 50–60 °C for REMAP primers. Amplifications were done in a thermocycler MC2 (DNA-Technology, Moscow, Russia). To control the purity of reagents, water was added to the reaction mixture instead of DNA (reactions C1- and C-). The experiments were done in triplicate.

### 2.4. Gel Electrophoresis of the Amplification Products

Fifteen microlitres of the reaction products were analysed in 2% agarose-TBE gels with EtBr. The DNA molecular size marker M 100 bp+2 Kb+3 Kb (12 fragments from 100 bp to 3000 bp, Sibenzyme, Novosibirsk, Russia) was used to measure the sizes of DNA fragments.

### 2.5. Quantitative Estimates of Genetic Diversity

To quantitatively estimate the genetic diversity, the data are presented as binary trait matrices, where the presence or absence of the same size PCR bands were assigned values of 1 or 0, respectively. The value of 1 was assigned only to bands of high intensity consistently detected in all experiments. To estimate the diversity, the binary data for each population were summed, and the most common values were used to build the matrices. 

The binary trait matrices were used to derive the difference matrices, with the Nei and Li genetic diversity (Gd) calculated as follows: *Gd(xy) = 1 − 2Nxy/(Nx + Ny),* where *Nx* is the number of fragments present in profile *x*, but absent in profile *y*; *Ny* is the number of fragments present in profile *y*, but absent in profile *x*; *Nxy* is the number of fragments present in both profiles [31].

The matrices obtained were used to build phylogenetic trees. The trees were built by the Unweighted Pair Group Method with Arithmetic Mean (UPGMA) [32] using Treecon 1.3b software [33]. To evaluate the confidence intervals of the trees, the bootstrap method with 100 samples was used [34]. 

## 3. Results

All primers yielded PCR bands amplified from plant genomic DNA. A total of 39 RAPD and 17 ISSR primers were used. Depending on the primer, the number of amplified fragments varied from 3 to 16, with their sizes varying from 200 to 2000 bp; the optimal annealing temperature was determined experimentally for each primer. The optimal primer pairs were designed for REMAP. Five REMAP pairs gave sharp and consistent profiles for lupin and seven REMAP pairs for hogweed (Supplementary Table S1).

### 3.1. Molecular Analyses of L. polyphyllus

**Figure 1:**
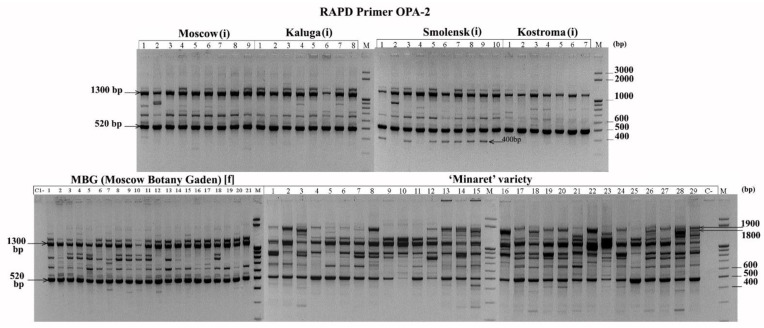
RAPD profile with the OPA-2 primer for *L. polyphyllus*. (i)—invasive population, [f]—feral population.

Some primers amplified fragments that were common for all analysed plants (RAPD: OPA-2—1300 bp and 520 bp, QR-5—730 bp, 450–850 bp; ISSR: MS1—400 bp; REMAP: MS4+TarI—210 bp, MS2+Thv19—170 bp). These fragments may represent nucleotide sequences specific to *L*. *polyphyllus*. Other primers amplified fragments specific for groups of plants (RAPD: OPA-2–large (about 2000 bp) fragments from the DNA of most plants of the ‘Minaret’ variety; a 400 bp fragment specific for Smolensk region plants; QR-5–1450; and 1500 bp fragments from the ‘Minaret’ variety; REMAP: MS7+Wis–900 bp from the feral population at MBG). Such fragments may be of interest as markers for those populations (Figure 1).

The genetic diversity between populations and the ‘Minaret’ variety varied from 0.196 to 0.341 and was 0.260, on average (Table 3). 

Additionally, the genetic diversity within invasive and feral populations and the ‘Minaret’ variety was calculated. To evaluate the genetic diversity within invasive population, the populations from Moscow, Kaluga, Smolensk, and Kostroma regions were pooled (34 samples). The amplified fragment profiles from 34 plant samples were presented as binary matrices and used to determine genetic diversity values (Supplementary Table S2). Depending on the method (RAPD, ISSR, or REMAP), the mean values of genetic diversity varied, but the general trend was the same: the invasive population had the highest values, and the ‘Minaret’ variety had the lowest values. With all the methods combined, the mean value of genetic diversity was 0.294 for the invasive population and 0.248 for the feral MBG population, which was 1.2-times lower than that for the invasive population. For the ‘Minaret’ variety, genetic diversity was 0.194, which was 1.5 times lower than that for the invasive population (Table 4). Notably, genetic diversity among populations (0.260) was less than the genetic diversity within invasive population (0.294).

**Figure 2:**
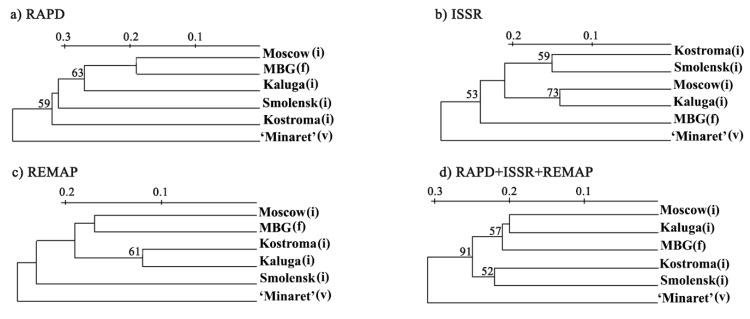
Phylogenetic trees of the analysed populations of *L. polyphyllus* derived from the data obtained by different methods. (**a**) Phylogenetic tree derived from the RAPD data, (**b**) Phylogenetic tree derived from the ISSR data, (**c**) Phylogenetic tree derived from the REMAP data, (**d**) Phylogenetic tree derived from the combination of RAPD, ISSR and REMAP data. The bootstrap values are given in %, (i)—invasive population, (f)—feral population, (v)—variety.

Phylogenetic trees were built by using individual methods (RAPD, ISSR, REMAP) and all methods combined (Figure 2).

Regardless of the method used, the ‘Minaret’ variety was considerably different from the other populations. RAPD and REMAP clustered together populations from the Moscow region and the feral population at MBG (Figure 2a,c). However, ISSR clustered Moscow and Kaluga region populations (Figure 2b). Altogether, the methods clustered the populations from Moscow and Kaluga regions with the feral MBG population; they also clustered Smolensk and Kostroma populations. This clustering almost reflects the geography of the populations. The Moscow Smolensk and Kaluga regions border each other, but the Kostroma region is far from them. The ‘Minaret’ variety was separated from these clusters (Figure 2). 

**Figure 3:**
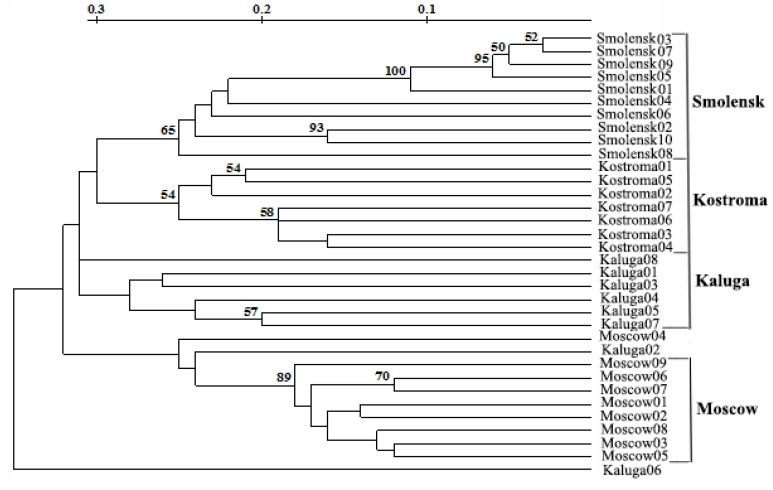
Phylogenetic tree of the invasive population of *L. polyphyllus* derived from the combination of RAPD, ISSR and REMAP data. The bootstrap values are given in %.

The phylogenetic tree of the invasive population had four clusters that almost coincided with the geographic origins of Moscow, Kaluga, Smolensk, and Kostroma populations (Figure 3).

The genetic diversity values within the feral MBG population (21 samples) and the ‘Minaret’ variety (29 samples) were calculated similarly (Supplementary Tables S3 and S4, respectively), and phylogenetic trees were built (data not shown).

### 3.2. Molecular Analyses of H. sosnowskyi

**Figure 4:**
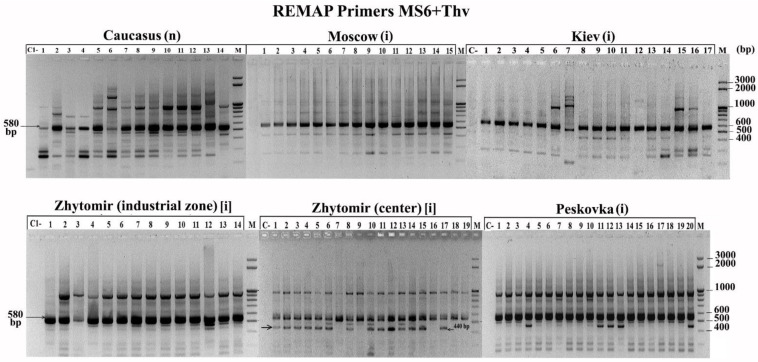
REMAP profile with the MS+Thv19 primers for *H. sosnowskyi*. (i)—invasive population, (n)—native population.

In hogweed, in contrast to lupin, only a few primers amplified monomorphic fragments typical for all plants and some populations (RAPD: QR-1—900 bp, QR-2—590 and 800 bp; ISSR: MS1—1500 bp; REMAP: MS6 + Thv19—580 bp). Interestingly, with primers MS6 + Thv19, a 440 bp fragment was detected, which is characteristic of plants growing in the centre of Zhytomir but absent in plants of the industrial zone of Zhytomyr (Figure 4).

The populations from industrial zones and the centre of Zhitomir were pooled (33 samples), and the genetic diversity between the populations of hogweed was significant, varying from 0.438 to 0.614, and was 0.539, on average (Table 5). 

The greatest genetic diversity was between populations from Moscow and Caucasus (0.614). This could be explained by the significant remoteness of the Moscow population and its evolution in the process of adaptation to a new habitat. The least genetic diversity was found between the Zhitomir and Peskovka populations (0.438). This could be explained by anthropogenic dispersal from one region to another. This is hardly a possible result of gene flow, since the distance between the Zhitomir and Peskovka is about 100 km (Supplementary Figure S2). The genetic diversity within each of the populations was calculated and varied for the Moscow region from 0.217 to 0.606, with an average of 0.398 (Supplementary Tables S5), for Peskovka from 0.224 to 0.678 with an average of 0.418 (Supplementary Tables S6), for Kiev from 0.265 to 0.622 with an average of 0.441 (Supplementary Tables S7), for the Zhitomir industrial zone from 0.216 to 0.640 with an average of 0.446 (Supplementary Tables S8), for Zhitomir centre from 0.248 to 0.629 with an average of 0.457 (Supplementary Tables S9), and within the native Caucasian population from 0.244 to 0.739 with an average of 0.502 (Supplementary Tables S10). There were no significant differences in genetic diversity in populations from different regions of Zhytomyr. The genetic diversity within the native Caucasian population was compared with that within the invasive population but was not significantly higher (Table 6). Unlike lupin, the genetic diversity between hogweed populations was greater than the genetic diversity within populations.

**Figure 5:**
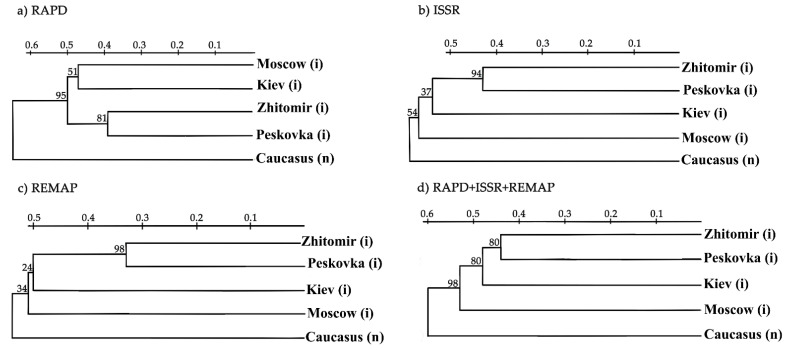
Phylogenetic trees of the analysed populations of *H. sosnowskyi* derived from the data obtained by different methods. (**a**) Phylogenetic tree derived from the RAPD data, (**b**) Phylogenetic tree derived from the ISSR data, (**c**) Phylogenetic tree derived from the REMAP data, (**d**) Phylogenetic tree derived from the combination of RAPD, ISSR and REMAP data. The bootstrap values are given in %, (i)—invasive population, (n)—native population.

Phylogenetic trees were built using individual methods (RAPD, ISSR, REMAP) and all methods combined (Figure 5).

Regardless of the method used, the Zhytomyr and Peskovka populations clustered together with high bootstrap values, indicating their close relationship (Figure 5). Hogweed from Zhitomir may have been introduced to Peskovka or vice versa. ISSR and REMAP clustered populations from Kiev, Zhitomir, and Peskovka that corresponded to their close geographical locations (Figure 5b,c). The use of RAPD combined Moscow and Kiev populations, but the dendrogram obtained by the combination of RAPD, ISSR, and REMAP revealed only one cluster with Zhitomir and Peskovka, although the Kiev population was close to them (Figure 5d). The Caucasian population was separated in all the dendrograms, suggesting a significant divergence between the native and invasive populations (Figure 5).

## 4. Discussion

### 4.1. Discussion of the Genetic Diversity within Populations of Lupin

RAPD, ISSR, and REMAP techniques were used to study the genetic diversity of various populations of *L*. *polyphyllus*. All techniques gave reproducible results with an adequate number of polymorphic and monomorphic fragments necessary for estimating the genetic diversity both within and between populations. As there are few investigations of *L*. *polyphyllus* by means of PCR markers, especially where genetic diversity was calculated [35,36], we considered studies of other species of *Lupinus*.

Qiu et al. [37] used short eight-nucleotide primers for studying *L*. *albus* L. Wolko et al. [38] identified polymorphisms using RAPD among four cultivars of *L*. *albus*, seven cultivars of *L*. *angustifolius* L., and twelve cultivars *of L*. *luteus*. Gilbert et al. [39] used ISSR to reveal genetic variability within and between *L*. *albus* accessions from a collection of lupin germplasm. Yorgancilar et al. [40] successfully used RAPD and ISSR to determine the genetic relationships among 20 Old World lupin genotypes of three lupin species (*L*. *albus*, *L*. *angustifolius*, and *L*. *luteus*). Using ISSR, Artyukhova [41] developed a technique for the identification of varieties of three lupin species: *L*. *angustifolius*, *L*. *hiteus*, and *L*. *albus*. Clements et al. [42] investigated 10 *L*. *angustifolius* genotypes using ISSR markers. Similarly, Guilengue et al. [43] analysed *L*. *mutabilis* using ISSR. Khapilina et al. [44] succeeded in using REMAP and other retrotransposon analysis methods to estimate the genetic diversity of *L*. *angustifolius*.

The reliability of ISSR, REMAP and, especially, RAPD is supported by the results obtained. Firstly, three phylogenetic trees of the analysed populations built upon RAPD, ISSR, and REMAP data showed the ‘Minaret’ variety is considerably divergent from the other populations (presumably due to its independent origin) (Figure 2). Secondly, the tree of the invasive population built on the combined data had four clusters that almost coincided with the locations of the respective populations, namely Moscow, Kaluga, Smolensk, and Kostroma regions (Figure 3). Thirdly, the genetic diversity values obtained by RAPD, ISSR, and REMAP for each population tightly correlated (Table 4).

The genetic diversity for the invasive population was the highest, and varied from 0.045 to 0.411 (Supplementary Table S2), with an average of 0.294 (Table 4). The genetic diversity among populations varied from 0.196 to 0.341 (Table 3) and was 0.260, on average, which was less than the genetic diversity within invasive population. Similar results have been previously published for other species of lupin. Vysniauskiene et al. [35] analysed 10 invasive populations of *L*. *polypyllus* in Lithuania with RAPD and found that Nei’s average genetic distance between populations was 0.148 ± 0.021, which was also less than the average genetic distance between individuals in the forest (0.290 ± 0.062) and field populations (0.229 ± 0.037). The authors observed significant genetic differentiation among populations in genetically heterogeneous seed stock, low gene flow between populations, and, possibly, in local adaptations [35]. Li et. al. [36] investigated 51 *L*. *polypyllus* populations in Finland using 13 polymorphic microsatellite loci. The authors found that the genetic variation among populations was 0.05; it was significantly higher (0.25) within populations. Pairwise F_ST_ values among populations ranged from 0.02 to 0.25 and the global F_ST_ value was 0.19, suggesting moderate levels of genetic differentiation. The authors suggested that it was the result of human-mediated dispersal with multiple introductions from different sources rather than the natural spread of *L. polyphyllus* from a single or few sources in Finland [36]. Oumer et al. [45] used ISSR to study four *L*. *albus* populations from two zones of Ethiopia and found that the genetic diversity was 0.223 for Merawi, 0.198 for Addis Kidam, 0.189 for Sekela, and 0.167 for Wembera: larger genetic diversity was found within rather than between populations. The authors supposed that high genetic diversity between populations might be the result of moderate gene flow and within the populations could be caused by the presence of preferential or diverse adaptive genes [45]. Similar data on the level of genetic distances were described by Mahfouze et al. [46], who estimated the genetic similarity among seven genotypes of *L*. *albus* using RAPD and ISSR, where the Nei genetic similarity index ranged from 0.74 to 0.88 (diversity from 0.12 to 0.26) [46].

In our study, the high level of genetic diversity within populations (among individual plants) was most likely associated with genetically heterogeneous seed stock and cross-pollination. The lower level of genetic diversity between populations was hardly a possible result of gene flow, since the distance between the Smolensk and Kostroma regions was about 700 km (Supplementary Figure S1), but more likely the result of seed source. Since lupin was cultivated for green mass or as an intermediate crop, the same standardised seed stock approved for cultivation was imported to different regions and could be the reason for the clustering of Smolensk and Kostroma populations on the dendrogram (Figure 2d).

There are studies where the values of genetic diversity within species are much higher than ours. Al Rawashdeh et al. [47] studied *L*. *pilosus* using RAPD and found its genetic similarity to range from 0.02 to 0.450 (diversity 0.550–0.980). The low similarity could have been due to a high, long diverging process in non-coding regions [47]. There are studies where the values of genetic diversity within species are more variable. Sbabou et al. [48] revealed the genetic similarity between and within Moroccan germplasm (*L*. *albus*, *L*. *angustifolius*, *L*. *cosentinii*, and *L*. *luteus*) by means of AFLP and ISSR markers. Similarity values were 0.70–0.82 (diversity 0.18–0.30) among accessions of *L*. *albus*, 0.24–0.61 (diversity 0.39–0.76) among accessions of *L*. *cosentinii*, and 0.68–0.88 (diversity 0.12–0.32) among accessions of *L*. *luteus* [48]. There are also studies where the values of genetic diversity within species are very low. Talhinhas et al. [49] used RAPD, ISSR, and AFLP to detect interspecific and intraspecific polymorphism in *Lupinus* spp. The intraspecific polymorphisms were revealed by AFLPs with similarity values of 0.908–0.955 (diversity 0.045–0.092) among *L*. *albus* accessions, 0.913–0.954 (0.046–0.087) in *L*. *angustifolius*, 0.920–0.988 (0.012–0.080) in *L*. *hispanicus*, 0.867–0.940 (0.060–0.033) in *L*. *luteus*, and 0.957–1.000 (0.043–0.000) in *L*. *mutabilis* [49].

Unfortunately, it was not possible to compare the genetic diversity of the invasive population in our study with the native population of *L*. *polypyllus* due to the lack of samples. Thus, it is unclear whether the invasive population has recovered after invasion and adaptation, or whether its genetic diversity is increasing. As far as we are aware, there are no published data on the genetic diversity or similarity of the native population of *L*. *polypyllus*.

In our research, in addition to the invasive and feral populations, the ‘Minaret’ variety was analysed. That this variety is not the source of the feral population is confirmed by all the dendrograms (Figure 2; unfortunately, information about the original variety has been lost). Although ‘Minaret’ cannot be a negative control for this feral population, low genetic diversity was expected as nearly all varieties have specific genotypes with selected characteristics which reduces their genetic diversity. Indeed, ‘Minaret’ had the lowest genetic diversity, varying from 0.099 to 0.288 (Supplementary Table S4), with 0.194, on average (Table 4).

The genetic diversity of the feral population (MBG) varied from 0.130 to 0.363 (Supplementary Table S3), with an average of 0.248 (Table 4), and was greater when compared to ‘Minaret’, but lower when compared to the invasive population. We believe that genetic diversity could have increased in the process of naturalization. The feral population, represented by the escaped variety, could be regarded as the beginning of the invasion. Additionally, the blue flowers characteristic for the wild species began to prevail. This may be due to gene flow by cross-pollination with other lupin plants growing nearby. 

Tkacheva [50] compared the biomorphological traits of feral (5 years of naturalisation) and Smolensk populations (30 and 40 years of naturalisation). The author found that the number of leaves and lateral flowering shoots per plant increased with a longer naturalisation period, but the number of seeds and plant height did not differ significantly. Although Ramula and Kalske [51] revealed that *L*. *polyphyllus* from the introduced populations were larger in size, they flowered less frequently and with fewer flowering shoots than plants from the native populations. Tkacheva [50] also studied the density of populations growing in forests and fields (Moscow and Smolensk regions). No significant differences in both populations were noted, indicating the ability of lupin to occupy biotopes with different levels of illumination. The author supposed that the success of *L*. *polyphyllus* as an invasive species could be explained by the increased competitive ability (EICA) hypothesis, which proposes that a larger size is selected due to intense intraspecific competition or reduced herbivore pressure in a new area [52].

The intermediate level of genetic diversity in the lupin feral population and high level in the invasive population discovered in our research, together with the other data revealed for the same populations (the increase of some biomorphological traits, and the ability to occupy new biotopes [50]) suggests the further expansion of *L. polyphyllus* invasion.

### 4.2. Discussion of the Genetic Diversity within Populations of Hogweed

Unfortunately, as with *L*. *polyphyllus*, there are few published investigations of *H*. *sosnowskyi* by means of PCR markers, especially where the values of genetic diversity have been calculated [53]. Therefore, again we analysed studies of other *Heracleum* spp. A study of *H*. *sosnowskyi* was performed previously using RAPD [54,55]. Strygina et al. [56] analysed the genus *Heracleum* using RAPD and ITS analysis, while noting its high genetic and morphological heterogeneity. 

We compared published data with our study, where the genetic diversity among the populations varied from 0.438 to 0.614 with an average of 0.539 (Table 5). Niinikoski and Korpelainen [57] investigated *H*. *mantegazzianum* from eight populations in Finland using microsatellite markers. The authors found that the range of pairwise F_ST_ values between populations was high (0.052–0.797), and the mean F_ST_ among all populations was 0.498, showing a significant difference. The authors observed differentiation between populations, resulting from multiple introductions combined with limited gene flow [57].

It is difficult to say why the average genetic diversity for lupin (0.260) was two times lower than for hogweed (0.539). Both species are diploid, *L. polyphyllus* with 2n = 48 [58] and *Heracleum* spp. with 2n = 22 [59]. It is probable that the hogweed genome contains more non-coding repeating regions, where as a rule, MAAP primers are annealing.

Unlike lupin, the genetic diversity among hogweed populations was greater (0.539) (Table 5) than that within the populations (0.398–0.502) (Table 6). This greater diversity might be explained by the large spread of values in genetic diversity within the population; for example, for the Caucasus from 0.244 to 0.739 (Supplementary Table S10), while the spread of values between populations was not so large 0.438 to 0.614 (Table 5). The lower genetic diversity within the populations also could be explained by the ability of *H*. *sosnowskyi* to self-pollinate. Walker et al. [60] investigated 13 *H*. *mantegazzianum* populations in northeast England using four nuclear microsatellite and one plastid marker. Similar to our research, the genetic differentiation between populations from different river catchments was higher (F_ST_ mean pairwise 0.28 ± 0.1) than that within populations from the same area (F_ST_ mean pairwise 0.11 ± 0.08). The overall F_ST_ mean pairwise was 0.24 ± 0.13. The authors suggested that the high genetic differentiation between populations may indicate a large initial founder population or multiple introductions. Within population, seeds of this species are dispersed in water, resulting in a relatively small dispersal area and the relatively small dispersal range of likely pollinators [60].

The genetic diversity within invasive populations varied from 0.398 to 0.457 with an average of 0.432 (Table 6); diversity was similar, but slightly less within the native Caucasian population with an average of 0.502 (Table 6). This suggests the successful naturalisation of invaders and reconstruction of their genetic diversity, probably by means of hybridisation and rapid evolution. In our study, similar genetic diversity values for invasive and native populations were observed. However, according to the literature, different cases have been described. Invasive populations may display higher genetic diversity than native populations and vice versa. Jahodová et al. [53] analysed three invasive *Heracleum* species using AFLPs. The genetic similarity (Pairwise Dice’s similarity coefficient) in *H*. *sosnowskyi* among all populations was 0.891, on average (diversity 0.109); within Europe, it was 0.901, on average (diversity 0.099), and within the native range, it was 0.908 (diversity 0.092). The authors noted that more within-taxon variation was detected in the invaded range (Europe) than in the region of the native distribution, probably driven by hybridisation and inbreeding. The results also indicate that multiple introductions, rapid evolution and drift are likely to have occurred [53]. Henry et al. [61] investigated 49 *H*. *mantegazzianum* populations from the western Swiss Alps and 11 Caucasian populations using eight nuclear microsatellite loci together with plastid DNA markers and sequences. F_ST_ mean pairwise genetic differentiation for nuclear microsatellite loci between invasive populations varied from 0.180 to 0.396 with an average of 0.315 and between native populations from 0.092 to 0.202 with an average of 0.162. The authors indicated that the high population differentiation observed in the invasive range compared to the native range could have been generated by sequential founder events. The species may have been introduced multiple times, possibly from disparate source populations [61]. Rijal et al. [62] studied 50 native and introduced populations of *H*. *persicum* with 27 microsatellite markers and estimated that F_ST_ mean pairwise genetic differentiation (averaged over the population) was lowest between England and Sweden (0.267 ± 0.006), and highest between Norway and Denmark (0.552 ± 0.005). The mean F_ST_ was lower in the native (0.25) compared to the introduced range (0.30), but the difference was marginal and nonsignificant [62].

Analysing other data such as the percentage of polymorphic loci, observed and expected heterozygosity, allelic richness and inbreeding coefficient, Henry et al. [61] and Rijal et al. [62] noted the loss of genetic diversity in the introduced ranges compared to native ranges. In our investigation, MAAP markers detected differences throughout the entire genome. As a rule, these markers are dominant and can detect only two alleles (present or absent fragment). At the same time, nuclear microsatellite markers, plastid, ribosomal, and mitochondrial DNA markers are locus specific and can detect several alleles, since their PCR fragments are sequenced. Therefore, the definition of ‘genetic diversity’ used for these techniques could be different. For our purposes, ‘genetic differentiation’ could be similar to ‘genetic diversity.’

According to the literature, invasive populations often have lower genetic diversity compared to the native population due to bottlenecking, genetic drift during colonisation, and founder effects [63]. However, in the territory of the former USSR, *H. sosnowskyi* was widely introduced at the state level. The Institute of Biology of the Komi Republic received *H. sosnowskyi* seeds from many botanical gardens and from native ranges (Nalchik). In the decade 1940–1950, accumulation of seeds and cultivation of this plant was of great importance in five main centres: Murmansk, Moscow, and Leningrad regions, the Republic of Komi, and Kabardino-Balkaria. For example, 140 hectares of *H. sosnowskyi* were planted in the Rzhevsky region, and 7600 kg of its seeds were sent to 60 addresses [64]. 

A rapid local adaptation and evolution in invasive ranges is characteristic of *H. Sosnowskyi.* The sizes of all parts of invasive plants of *H*. *sosnowsky* are larger than plants of native populations: the height of the stem (1–1.5 m in the Caucasus and 2–4 m in Europe) and the size of the seeds (in Caucasian species, 9 mm long and 6 mm wide; in Europe, 15 mm long and 8 mm wide). In addition, Caucasian plants grow in forests and meadows, whereas plants of the invaded range are photophilous and mainly found in open habitats [65].

A high level of genetic diversity in hogweed invasive populations, similar to the native Caucasion population, was found in our research, suggesting reconstruction of their genetic diversity. This high level of genetic diversity, together with data suggesting the increase of some biomorphological traits, the ability to occupy new biotopes [65], and to hybridize with the native *H. sibiricum* and *H. spondyllium* and other introduced hogweeds [66] reported in the literature, could drive the further expansion of *H. sosnowskyi* invasion.

## 5. Conclusions

Both *L. polyphyllus* and *H. sosnowskyi* have been used in agriculture (lupin since 1922 [11], hogweed since 1947 [16]). Many seeds of both crops were imported from different ranges, including their natural habitats, so that multiple introductions resulted in a large number of founders. It is probable that the genetic diversity did not initially decrease. Moreover, the genetic diversity could have increased as a result of genetic exchanges between populations originating from different regions. Therefore, in this case we cannot speak to the genetic paradox of invasion, because genetic variation in populations is not lower than the native source population and has not passed through a bottleneck [67]. Where varieties were bred, genetic diversity was likely reduced, but after naturalization to a new habitat, diversity in feral populations began to increase again. Therefore, our results suggest that anthropogenic dispersal may be a major factor contributing to the successful invasion of *L. polyphyllus* and *H. sosnowskyi*, together with their individual characteristics, which include environmental tolerance, high fertility, phenotypic plasticity, cold resistance, and the ability to occupy new biotopes. Both species have turned into transformers and supplanted the native species, significantly reducing the diversity of biogeocenoses [65,68]. Thus, these species require further study to successfully control their invasion.

## Figures and Tables

**Table 1:** Phenotypes, collection sites and population types of the *L. polyphyllus* samples.

Collection Site	Population Type	Number of Samples	Colour of Flowers	Latitude	Longitude
MBG (Moscow Botany Garden) territory, Russia	Feral, running wild for about five years	21	blue, pink, white	55°83′52.9″	37°60′92.0″
Moscow region, Russia	Invasive, grows for decades	9	blue, pink, white	55°38′64.6″	39°18′24.1″
Kaluga region, Russia		8	blue	54°50′91.3″	36°21′24.6″
Kostroma region, Russia		7	blue	57°81′45.7″	40°98′37.7″
Smolensk region, Russia		10	blue, pink	54°72′77.5″	32°99′65.3″
Plants grown from the seeds in the greenhouse	‘Minaret’ variety	29	blue, pink, white		

**Table 2:** Phenotypes, collection sites and population types of the *H. sosnowskyi* samples.

Collection Site	Population Type	Number of Samples	Colour of Flowers	Latitude	Longitude
Moscow, Russia	Invasive, grows for decades	15	white	55°57′41.7″	37°55′56.2″
Peskovka, Ukraine		20	white	50°70′69.1″	29°58′15.3″
Kiev, Ukraine		17	white	50°46′12.2″	30°55′63.1″
Zhitomir (industrial), Ukraine		14	white	50°24′14.3″	28°74′12.8″
Zhitomir (center), Ukraine		19	white	50°25′29.0″	28°65′14.9″
Caucasus, Russia	native	14	white	43°77′66.0″	43°28′72.5″

**Table 3:** Genetic diversity between the analysed populations of L. polyphyllus calculated according to Nei and Li using the combination of methods RAPD, ISSR and REMAP.

Collection Site	Kostroma (i)	Smolensk (i)	Moscow (i)	Kaluga (i)	MBG (f)	‘Minaret’ Variety
**Kostroma (i)**	0.000					
**Smolensk (i)**	0.216	0.000				
**Moscow (i)**	0.209	0.260	0.000			
**Kaluga (i)**	0.231	0.258	0.196	0.000		
**MBG (f)**	0.269	0.284	0.198	0.230	0.000	
**‘Minaret’ Variety**	0.302	0.315	0.318	0.341	0.276	0.000

(i)—invasive population, (f)—feral population.

**Table 4:** The mean values of genetic diversity (GD) for *L. polyphyllus* invasive and feral populations and the ‘Minaret’ variety obtained by RAPD, ISSR, REMAP and the combination thereof; (RATIO), the ratio of the genetic diversity of the invasive population over the genetic diversity of the given population.

Collection Site	RAPD		ISSR		REMAP		RAPD+ISSR+REMAP	
	GD	RATIO	GD	RATIO	GD	RATIO	GD	RATIO
**Invasive population**	0.322		0.253		0.328		0.294	
**Feral MBG Population**	0.234	1.4	0.238	1.1	0.290	1.1	0.248	1.2
**‘Minaret’ Variety**	0.205	1.6	0.171	1.5	0.220	1.5	0.194	1.5

**Table 5:** Genetic diversity between the analysed populations of H. sosnowskyi calculated according to Nei and Li using the combination of methods RAPD, ISSR and REMAP.

Collection site	Caucasus (n)	Moscow (i)	Kiev (i)	Zhitomir (i)	Pescovka (i)
**Caucasus (n)**	0.000				
**Moscow (i)**	0.614	0.000			
**Kiev (i)**	0.611	0.512	0.000		
**Zhitomir (i)**	0.591	0.530	0.493	0.000	
**Pescovka (i)**	0.569	0.562	0.466	0.438	0.000

(i)—invasive population, (n)—native population.

**Table 6:** The mean values of genetic diversity (GD), and their maximum and minimum values for *H. sosnowskyi* invasive and native populations obtained by combination of methods RAPD, ISSR and REMAP.

Populations	RAPD+ISSR+REMAP			
	Min GD	Max GD	Mean GD	Difference of GD between Native and Invasive Populations
**Moscow (i)**	0.217	0.606	0.398	0.104
**Peskovka (i)**	0.224	0.678	0.418	0.084
**Kiev (i)**	0.265	0.622	0.441	0.061
**Zhitomir (industrial) (i)**	0.216	0.640	0.446	0.056
**Zhitomir (center) (i)**	0.248	0.629	0.457	0.045
**The average Value of Genetic Diversity within Invasive Populations**	0.234	0.635	0.432	0.070
**Caucasus (n)**	0.244	0.739	0.502	

(i)—invasive population, (n)—native population.

## Data Availability

The data presented in this study are available upon request from the corresponding author.

## Supplementary Materials

The following materials are available online at https://www.mdpi.com/article/10.3390/biology10111094/s1, Figure S1: Map of the lupin sampled populations, Figure S2: Map of the hogweed sampled populations, Table S1: The primers used in the experiments, Table S2: Genetic diversity within lupin invasive population obtained by combination of RAPD, ISSR and REMAP data, Table S3: Genetic diversity within lupin feral MBG population obtained by combination of RAPD, ISSR and REMAP data, Table S4: Genetic diversity within the ‘Minaret’ variety obtained by combination of RAPD, ISSR and REMAP data, Table S5: Genetic diversity within hogweed Moscow invasive population obtained by combination of RAPD, ISSR and REMAP data, Table S6: Genetic diversity within hogweed Peskovka invasive population obtained by combination of RAPD, ISSR and REMAP data, Table S7: Genetic diversity within hogweed Kiev invasive population obtained by combination of RAPD, ISSR and REMAP data, Table S8: Genetic diversity within hogweed Zhitomir industrial zone invasive population obtained by combination of RAPD, ISSR and REMAP data, Table S9: Genetic diversity within hogweed Zhitomir centre invasive population obtained by combination of RAPD, ISSR and REMAP data, Table S10: Genetic diversity within hogweed Caucasus native population obtained by combination of RAPD, ISSR and REMAP data.
