# Supplementary material for: Genetic Diversity in Invasive Populations of Lupinus polyphyllus Lindl. and Heracleum sosnowskyi Manden
Source: Biology (Basel). 2021 Oct 25;10(11):1094. doi: 10.3390/biology10111094 (PMC8614902; doi:10.3390/biology10111094)
Supplement: Supplementary file 1 [file biology-10-01094-s001.zip › biology-1367406-supplementary.pdf]

Figure S1: Map of the lupin sampled populations.

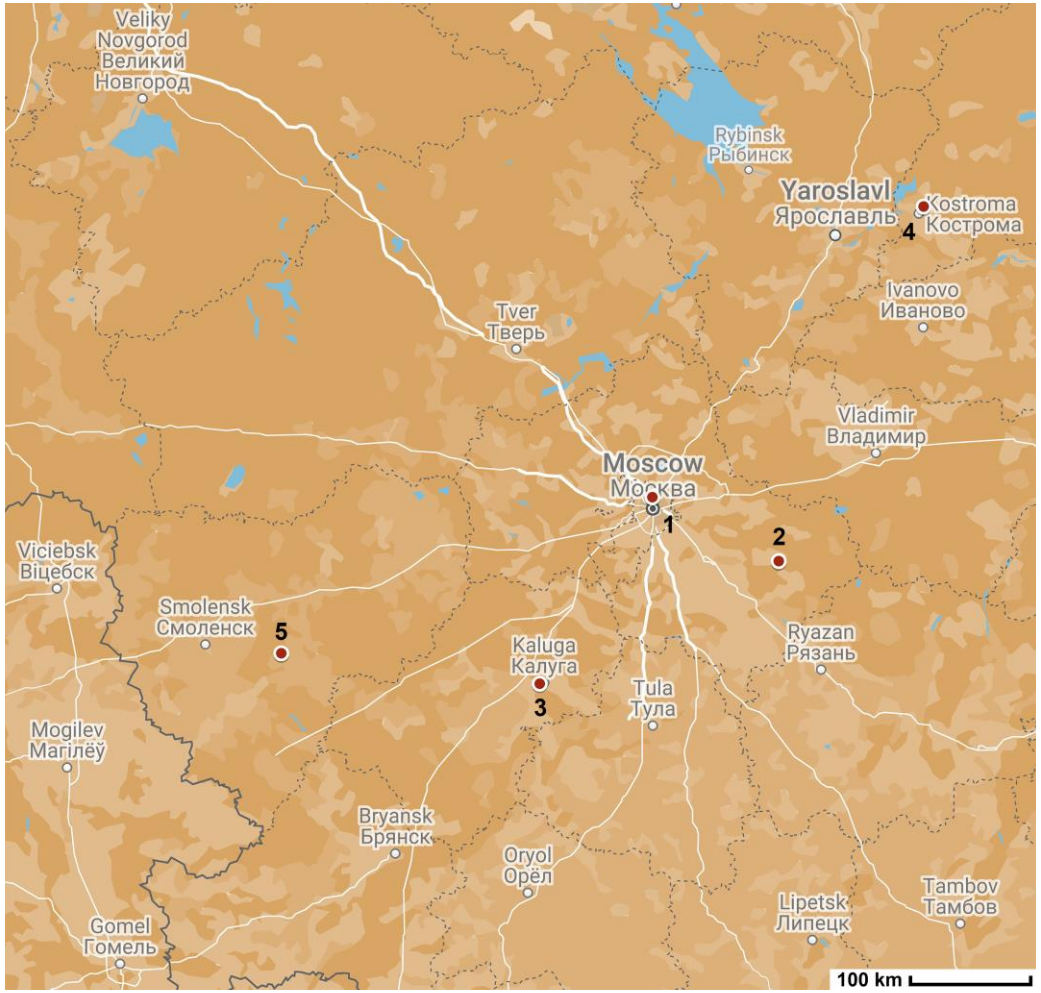

1. MBG (Moscow Botany Garden) territory, 2. Moscow region, 3. Kaluga region, 4. Kostroma region, 5 Smolensk region

Figure S2: Map of the hogweed sampled populations.

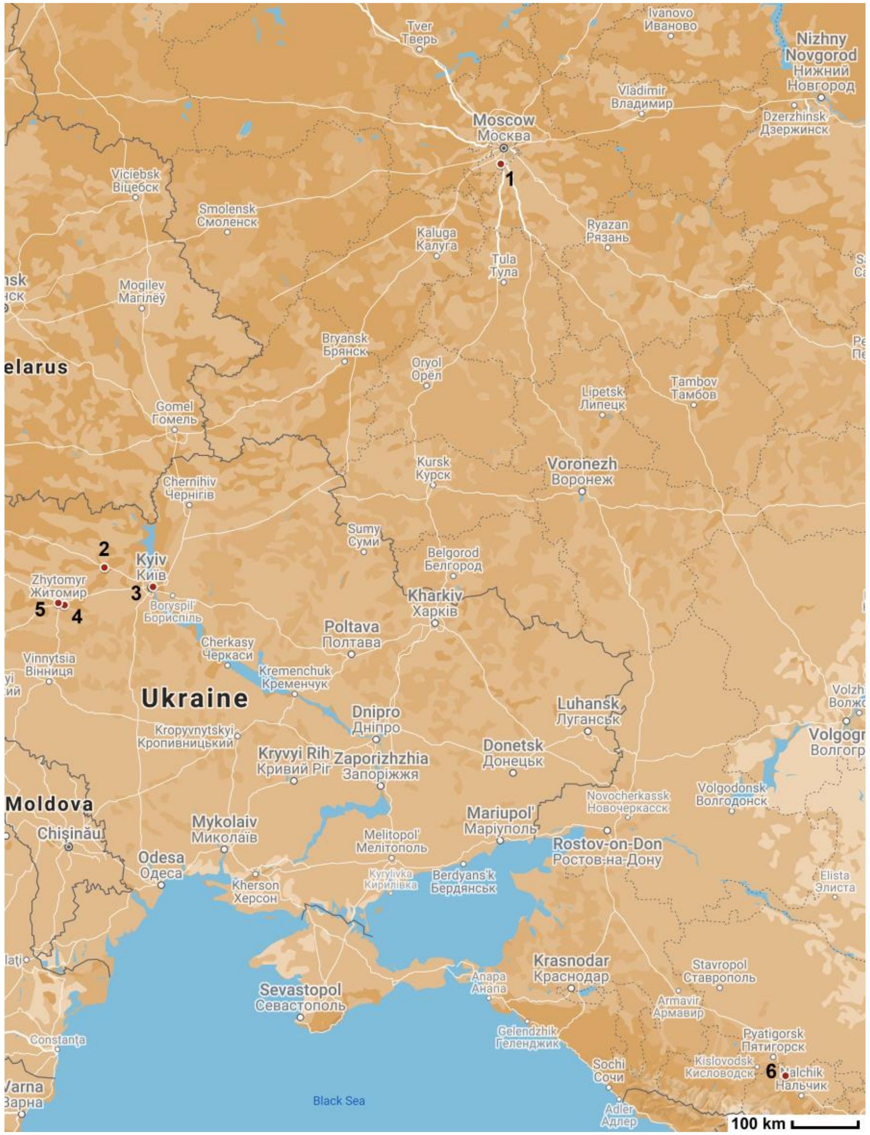

1. Moscow, Russia; 2. Peskovka, Ukraine; 3. Kiev, Ukraine; 4. Zhitomir (industrial), Ukraine; 5. Zhitomir (center), Ukraine; 6. Caucasus, Russia

Table S1: The primers used in the experiment.

technique	primer	sequence (5′ – 3′)	T °C	reference
**RAPD**	**A-12**	ATC-GCA-CAC-T	**37**	[1]
	**AD-04**	GTA-GGC-CTC-A	**37**	
	**AE-07**	GTG-TCA-GTG-G	**37**	
	**AE-13**	TGT-GGA-CTG-G	**37**	
	**Ag-1**	AGG-TCA-CTG-A	**37**	
	**AH-29**	TGG-TGA-CTG-A	**37**	
	**AH-30**	TGG-TCA-CTG-T	**37**	
	**E-16**	GGT-GAC-TGT-G	**37**	
	**M-06**	CTG-GGC-AAC-T	**37**	
	**M-07**	CCG-TGA-CTC-A	**37**	
	**M-14**	AGG-GTC-GTT-C	**37**	
	**NO-15**	CAG-CGA-CTG-T	**37**	
	**O-07**	CAG-CAC-TGA-C	**37**	
	**OPA-02**	TGC-CGA-GCT-G	**37**	
	**OPA-04**	AGT-CAG-CCA-C	**37**	
	**OPC-09**	CTC-ACC-GTC-C	**37**	
	**Q-09**	GGC-TAA-CCG-A	**37**	
	**Q-20**	TCG-CCC-AGT-C	**37**	
	**R-03**	ACA-CAG-AGG-G	**37**	
	**R-06**	GTC-TAC-GGC-A	**37**	
	**R-11**	GTA-GCC-GTC-T	**37**	
	**R-12**	ACA-GGT-GCG-T	**37**	
	**V-03**	CTC-CCT-GCA-A	**37**	
	**V-20**	CAG-CAT-GGT-C	**37**	
	**X-01**	CTG-GGC-ACG-A	**37**	
	**X-15**	CAG-ACA-AGC-C	**37**	
	**G-1**	CCT-GTT-AGC-C	**37**	
	**Leb-10**	AGC-CGC-AGC-T	**37**	
	**P-10**	AGG-CGG-GTA-C	**37**	
	**P-11**	AGG-CGG-GAA-CG	**37**	
	**P-474**	AGG-CGG-GAA-C	**37**	
	**P-318**	CGG-AGA-GCG-A	**37**	
	**P-340**	GAG-AGG-CAC-C	**37**	
	**P-450**	CGG-AGA-GCC-C	**37**	
	**QR-1**	CGG-TCA-CTG-T	**37**	
	**QR-2**	CGG-CCA-CTG-T	**37**	
	**QR-3**	CGG-CCC-CTG-T	**37**	
	**QR-4**	CGG-CCC-CGG-T	**37**	
	**QR-5**	CGG-CCC-CGG-C	**37**	
**ISSR**	**M-1**	$(AC)_8CG$	**55**	
	**M-2**	$(AC)_8C/CT$	**55**	
	**M-6**	$(CAC)_5$	**50**	
	**M-7**	$(CAG)_5$	**50**	
	**M-8**	$(GTG)_5$	**50**	
	**M-9**	$(GACAC)_4$	**40**	
	**M-10**	(CA) 6A/GG	**45**	
	**M-11**	(CA) 6A/G	**45**	
	**M-12**	(CA) 6RY	**50**	
	**M-13**	$(AGC)_3Y$	**40**	
	**MS1**	$(CA)_8AC$	**37**	[2]

	**MS2**	(AG)$_8$CA	**45**	[3]
	**MS3**	(AG)$_8$TA	**45**	
	**MS4**	(AC)$_9$T	**55**	[4]
	**MS5**	(AGC)$_6$T	**50**	
	**MS6**	(GAG)$_6$C	**60**	[5]
	**MS7**	(GA)$_8$CAG	**55**	
**LTR**	**Wis-2-1-A**	CTA-GGG-CAT-AAT-TCC-AAC-AA		[6]
	**TarI**	CTC-CCA-GTT-GAC-CAA-CAA		
	**Tagermina**	AGA-GGA-GGA-TAT-CCC-AAC-CAT		
	**Thv19**	GCC-CAA-CCG-ACC-AGG-TTG-TTA-CAG		
**REMAP for lupine**	**MS7+Wis-2-1-A**	(GA)$_8$CAG CTA-GGG-CAT-AAT-TCC-AAC-AA	**55**	ISSR+LTR
	**MS4+TarI**	(AC)$_9$T CTC-CCA-GTT-GAC-CAA-CAA	**50**	
	**MS1+Tagermina**	(CA)$_8$AC AGA-GGA-GGA-TAT-CCC-AAC-CAT	**50**	
	**MS3+Tagermina**	(AG)$_8$TA AGA-GGA-GGA-TAT-CCC-AAC-CAT	**55**	
	**MS2+Thv19**	(AG)$_8$CA GCC-CAA-CCG-ACC-AGG-TTG-TTA-CAG	**50**	
**REMAP for hogweed**	**MS1+Wis-2-1-A**	(CA)$_8$AC CTA-GGG-CAT-AAT-TCC-AAC-AA	**55**	ISSR+LTR
	**MS1+TarI**	(CA)$_8$AC CTC-CCA-GTT-GAC-CAA-CAA	**55**	
	**MS2+TarI**	(AG)$_8$CA CTC-CCA-GTT-GAC-CAA-CAA	**55**	
	**MS3+Thv19**	(AG)$_8$TA GCC-CAA-CCG-ACC-AGG-TTG-TTA-CAG	**50**	
	**MS3+Tagermina**	(AG)$_8$TA AGA-GGA-GGA-TAT-CCC-AAC-CAT	**55**	
	**MS4+ TarI**	(AC)$_9$T CTC-CCA-GTT-GAC-CAA-CAA	**55**	
	**MS6+Thv19**	(GAG)$_6$C GCC-CAA-CCG-ACC-AGG-TTG-TTA-CAG	**60**	

Table S2: Genetic diversity within lupin invasive population obtained by combinations of RAPD, ISSR and REMAP data

	Kostr1	Kostr2	Kostr3	Kostr4	Kostr5	Kostr6	Kostr7	Smol1	Smol2	Smol3	Smol4	Smol5	Smol6	Smol7	Smol8	Smol9	Smol10
**Kostr1**	0.000																
**Kostr2**	0.221	0.000															
**Kostr3**	0.240	0.200	0.000														
**Kostr4**	0.241	0.281	0.164	0.000													
**Kostr5**	0.207	0.237	0.233	0.257	0.000												
**Kostr6**	0.254	0.214	0.186	0.189	0.247	0.000											
**Kostr7**	0.268	0.262	0.198	0.188	0.285	0.188	0.000										
**Smol1**	0.259	0.301	0.285	0.321	0.313	0.313	0.367	0.000									
**Smol2**	0.284	0.301	0.261	0.310	0.325	0.337	0.304	0.269	0.000								
**Smol3**	0.266	0.295	0.244	0.314	0.306	0.282	0.333	0.104	0.252	0.000							
**Smol4**	0.278	0.306	0.256	0.314	0.318	0.329	0.321	0.252	0.276	0.212	0.000						
**Smol5**	0.237	0.266	0.227	0.285	0.276	0.253	0.314	0.126	0.234	0.069	0.218	0.000					
**Smol6**	0.325	0.329	0.301	0.325	0.304	0.391	0.359	0.247	0.234	0.230	0.230	0.224	0.000				
**Smol7**	0.249	0.288	0.239	0.296	0.299	0.276	0.325	0.102	0.246	0.034	0.195	0.045	0.212	0.000			
**Smol8**	0.337	0.352	0.291	0.337	0.306	0.341	0.393	0.265	0.265	0.249	0.237	0.243	0.268	0.232	0.000		
**Smol9**	0.277	0.294	0.243	0.314	0.293	0.281	0.333	0.113	0.250	0.042	0.210	0.076	0.241	0.053	0.247	0.000	
**Smol10**	0.298	0.267	0.228	0.322	0.361	0.349	0.366	0.284	0.160	0.219	0.231	0.214	0.250	0.214	0.267	0.217	0.000
**Mosc1**	0.289	0.304	0.257	0.258	0.304	0.315	0.295	0.322	0.333	0.315	0.348	0.276	0.291	0.286	0.326	0.315	0.322
**Mosc2**	0.356	0.292	0.266	0.311	0.314	0.326	0.341	0.345	0.333	0.349	0.360	0.318	0.313	0.318	0.337	0.349	0.333
**Mosc3**	0.369	0.372	0.310	0.322	0.325	0.337	0.317	0.358	0.370	0.337	0.361	0.341	0.325	0.329	0.384	0.325	0.345
**Mosc4**	0.342	0.383	0.292	0.317	0.321	0.384	0.377	0.395	0.368	0.358	0.321	0.337	0.360	0.350	0.370	0.321	0.316
**Mosc5**	0.329	0.311	0.261	0.274	0.264	0.264	0.302	0.317	0.353	0.299	0.368	0.270	0.358	0.281	0.367	0.287	0.329
**Mosc6**	0.349	0.318	0.257	0.303	0.306	0.283	0.310	0.373	0.337	0.341	0.376	0.333	0.341	0.333	0.330	0.341	0.360
**Mosc7**	0.333	0.315	0.277	0.311	0.314	0.303	0.318	0.310	0.298	0.291	0.360	0.274	0.325	0.285	0.326	0.291	0.322
**Mosc8**	0.337	0.352	0.270	0.271	0.318	0.318	0.298	0.337	0.337	0.307	0.341	0.289	0.329	0.289	0.341	0.295	0.337
**Mosc9**	0.337	0.284	0.246	0.292	0.341	0.283	0.321	0.301	0.325	0.318	0.353	0.277	0.341	0.288	0.295	0.306	0.337
**Kal1**	0.361	0.272	0.314	0.349	0.306	0.318	0.382	0.288	0.325	0.306	0.282	0.276	0.354	0.287	0.306	0.281	0.349
**Kal2**	0.273	0.254	0.238	0.275	0.277	0.277	0.267	0.333	0.308	0.301	0.277	0.282	0.299	0.294	0.349	0.288	0.285
**Kal3**	0.282	0.310	0.260	0.273	0.287	0.251	0.349	0.293	0.317	0.275	0.310	0.269	0.346	0.269	0.322	0.286	0.318
**Kal4**	0.282	0.263	0.296	0.358	0.299	0.338	0.368	0.307	0.320	0.338	0.338	0.304	0.351	0.329	0.375	0.325	0.346
**Kal5**	0.329	0.266	0.307	0.318	0.310	0.299	0.349	0.317	0.317	0.322	0.333	0.303	0.358	0.303	0.333	0.322	0.318
**Kal6**	0.296	0.362	0.358	0.358	0.363	0.363	0.432	0.359	0.320	0.350	0.338	0.329	0.391	0.329	0.350	0.312	0.308
**Kal7**	0.313	0.257	0.289	0.302	0.329	0.354	0.333	0.287	0.274	0.305	0.354	0.298	0.316	0.286	0.353	0.317	0.313
**Kal8**	0.310	0.302	0.287	0.345	0.337	0.314	0.366	0.309	0.321	0.290	0.325	0.249	0.363	0.272	0.314	0.313	0.286

	Mosc1	Mosc2	Mosc3	Mosc4	Mosc5	Mosc6	Mosc7	Mosc8	Mosc9	Kal1	Kal2	Kal3	Kal4	Kal5	Kal6	Kal7	Kal8
**Kostr1**																	
**Kostr2**																	
**Kostr3**																	
**Kostr4**																	
**Kostr5**																	
**Kostr6**																	
**Kostr7**																	
**Smol1**																	
**Smol2**																	
**Smol3**																	
**Smol4**																	
**Smol5**																	
**Smol6**																	
**Smol7**																	
**Smol8**																	
**Smol9**																	
**Smol10**																	
**Mosc1**	0.000																
**Mosc2**	0.140	0.000															
**Mosc3**	0.167	0.184	0.000														
**Mosc4**	0.247	0.280	0.241	0.000													
**Mosc5**	0.168	0.184	0.121	0.227	0.000												
**Mosc6**	0.163	0.191	0.174	0.247	0.164	0.000											
**Mosc7**	0.172	0.178	0.195	0.256	0.173	0.124	0.000										
**Mosc8**	0.134	0.149	0.131	0.200	0.122	0.162	0.127	0.000									
**Mosc9**	0.185	0.169	0.174	0.272	0.198	0.170	0.180	0.162	0.000								
**Kal1**	0.337	0.314	0.349	0.396	0.310	0.341	0.337	0.330	0.295	0.000							
**Kal2**	0.243	0.216	0.224	0.303	0.271	0.266	0.240	0.244	0.231	0.289	0.000						
**Kal3**	0.319	0.341	0.306	0.338	0.291	0.299	0.307	0.345	0.276	0.263	0.281	0.000					
**Kal4**	0.345	0.346	0.410	0.397	0.366	0.400	0.370	0.387	0.363	0.312	0.294	0.316	0.000				
**Kal5**	0.308	0.307	0.283	0.411	0.292	0.277	0.330	0.311	0.288	0.230	0.282	0.280	0.255	0.000			
**Kal6**	0.404	0.382	0.371	0.342	0.378	0.399	0.370	0.386	0.362	0.338	0.333	0.304	0.320	0.293	0.000		
**Kal7**	0.280	0.278	0.325	0.399	0.333	0.329	0.314	0.318	0.269	0.280	0.250	0.273	0.232	0.202	0.377	0.000	
**Kal8**	0.322	0.345	0.357	0.354	0.306	0.337	0.310	0.303	0.314	0.314	0.297	0.271	0.359	0.306	0.371	0.301	0.000

Table S3: Genetic diversity within lupin feral MBG population obtained by combination of RAPD, ISSR and REMAP data

	MBG1	MBG2	MBG3	MBG4	MBG5	MBG6	MBG7	MBG8	MBG9	MBG10	MBG11	MBG12	MBG13	MBG14	MBG15	MBG16	MBG17	MBG18	MBG19	MBG20	MBG21
**MBG1**	0.000																				
**MBG2**	0.275	0.000																			
**MBG3**	0.202	0.205	0.000																		
**MBG4**	0.276	0.217	0.178	0.000																	
**MBG5**	0.282	0.321	0.256	0.311	0.000																
**MBG6**	0.287	0.275	0.236	0.289	0.338	0.000															
**MBG7**	0.283	0.239	0.249	0.250	0.292	0.309	0.000														
**MBG8**	0.214	0.275	0.214	0.252	0.231	0.350	0.214	0.000													
**MBG9**	0.237	0.239	0.191	0.226	0.280	0.333	0.202	0.191	0.000												
**MBG10**	0.238	0.301	0.250	0.277	0.243	0.262	0.273	0.238	0.261	0.000											
**MBG11**	0.246	0.213	0.166	0.235	0.264	0.244	0.189	0.234	0.167	0.222	0.000										
**MBG12**	0.279	0.234	0.256	0.210	0.363	0.292	0.254	0.267	0.254	0.293	0.196	0.000									
**MBG13**	0.275	0.276	0.240	0.241	0.296	0.263	0.250	0.240	0.227	0.288	0.202	0.223	0.000								
**MBG14**	0.230	0.254	0.184	0.160	0.309	0.239	0.229	0.218	0.218	0.265	0.182	0.180	0.130	0.000							
**MBG15**	0.237	0.216	0.168	0.214	0.317	0.222	0.213	0.237	0.247	0.297	0.200	0.232	0.193	0.173	0.000						
**MBG16**	0.269	0.271	0.293	0.272	0.303	0.308	0.291	0.317	0.326	0.333	0.276	0.310	0.271	0.295	0.244	0.000					
**MBG17**	0.257	0.281	0.269	0.259	0.350	0.305	0.211	0.269	0.256	0.317	0.242	0.285	0.292	0.260	0.222	0.322	0.000				
**MBG18**	0.254	0.267	0.220	0.198	0.333	0.289	0.231	0.198	0.231	0.278	0.207	0.204	0.178	0.148	0.187	0.284	0.261	0.000			
**MBG19**	0.264	0.243	0.207	0.195	0.309	0.288	0.218	0.276	0.218	0.325	0.215	0.258	0.232	0.200	0.173	0.295	0.171	0.169	0.000		
**MBG20**	0.244	0.234	0.209	0.210	0.338	0.280	0.198	0.209	0.209	0.232	0.218	0.250	0.211	0.169	0.209	0.275	0.240	0.182	0.169	0.000	
**MBG21**	0.273	0.238	0.236	0.288	0.346	0.286	0.212	0.273	0.235	0.325	0.174	0.243	0.262	0.240	0.188	0.305	0.256	0.241	0.228	0.254	0.000

Table S4: Genetic diversity within lupin 'Minaret' variety obtained by combination of RAPD, ISSR and REMAP data

	Min1	Min2	Min3	Min4	Min5	Min6	Min7	Min8	Min9	Min10	Min11	Min12	Min13	Min14	Min15	Min16	Min17	Min18	Min19	Min20	Min21	Min22	Min23	Min24	Min25	Min26	Min27	Min28	Min29
**Min1**	0.000																												
**Min2**	0.181	0.000																											
**Min3**	0.169	0.163	0.000																										
**Min4**	0.202	0.219	0.195	0.000																									
**Min5**	0.154	0.193	0.171	0.202	0.000																								
**Min6**	0.224	0.193	0.170	0.214	0.178	0.000																							
**Min7**	0.179	0.149	0.172	0.181	0.148	0.121	0.000																						
**Min8**	0.207	0.189	0.189	0.209	0.207	0.195	0.186	0.000																					
**Min9**	0.195	0.236	0.200	0.173	0.195	0.207	0.126	0.214	0.000																				
**Min10**	0.195	0.165	0.165	0.174	0.173	0.172	0.140	0.191	0.153	0.000																			
**Min10**	0.195	0.200	0.166	0.186	0.174	0.149	0.130	0.202	0.155	0.145	0.000																		
**Min12**	0.168	0.234	0.162	0.183	0.170	0.217	0.195	0.188	0.163	0.224	0.176	0.000																	
**Min13**	0.146	0.130	0.141	0.161	0.161	0.182	0.151	0.156	0.200	0.177	0.178	0.163	0.000																
**Min14**	0.123	0.164	0.107	0.149	0.183	0.205	0.151	0.189	0.176	0.166	0.156	0.128	0.099	0.000															
**Min15**	0.214	0.184	0.207	0.240	0.235	0.272	0.227	0.220	0.257	0.244	0.243	0.243	0.117	0.173	0.000														
**Min16**	0.200	0.193	0.193	0.226	0.200	0.200	0.191	0.230	0.207	0.207	0.184	0.229	0.170	0.182	0.214	0.000													
**Min17**	0.250	0.241	0.241	0.265	0.269	0.248	0.238	0.267	0.232	0.269	0.217	0.216	0.227	0.215	0.263	0.223	0.000												
**Min18**	0.165	0.182	0.148	0.191	0.146	0.200	0.124	0.173	0.160	0.161	0.151	0.170	0.138	0.138	0.225	0.166	0.247	0.000											
**Min19**	0.198	0.157	0.146	0.189	0.187	0.175	0.167	0.238	0.205	0.205	0.182	0.179	0.115	0.126	0.178	0.186	0.232	0.143	0.000										
**Min20**	0.195	0.189	0.189	0.175	0.185	0.184	0.143	0.180	0.214	0.191	0.180	0.211	0.135	0.157	0.165	0.173	0.217	0.141	0.118	0.000									
**Min21**	0.197	0.215	0.190	0.175	0.233	0.235	0.176	0.241	0.154	0.155	0.157	0.215	0.202	0.155	0.236	0.198	0.208	0.186	0.207	0.193	0.000								
**Min22**	0.216	0.220	0.209	0.207	0.194	0.205	0.207	0.211	0.212	0.189	0.178	0.233	0.165	0.187	0.184	0.159	0.252	0.182	0.169	0.178	0.167	0.000							
**Min23**	0.205	0.232	0.186	0.172	0.183	0.227	0.173	0.233	0.165	0.200	0.133	0.163	0.165	0.165	0.251	0.205	0.264	0.149	0.169	0.189	0.190	0.176	0.000						
**Min24**	0.208	0.224	0.212	0.235	0.253	0.195	0.198	0.274	0.266	0.227	0.226	0.275	0.176	0.176	0.246	0.159	0.232	0.183	0.181	0.179	0.192	0.212	0.224	0.000					
**Min25**	0.241	0.233	0.233	0.254	0.204	0.216	0.184	0.246	0.236	0.247	0.223	0.246	0.198	0.220	0.264	0.228	0.316	0.170	0.191	0.178	0.252	0.209	0.164	0.224	0.000				
**Min26**	0.202	0.207	0.160	0.205	0.225	0.214	0.170	0.198	0.210	0.234	0.198	0.183	0.172	0.172	0.216	0.238	0.277	0.145	0.177	0.198	0.200	0.230	0.172	0.235	0.195	0.000			
**Min27**	0.190	0.195	0.149	0.205	0.158	0.168	0.148	0.209	0.198	0.209	0.164	0.160	0.140	0.128	0.216	0.202	0.200	0.146	0.144	0.165	0.212	0.207	0.184	0.210	0.230	0.170	0.000		
**Min28**	0.205	0.277	0.164	0.207	0.194	0.182	0.207	0.211	0.235	0.246	0.211	0.198	0.187	0.165	0.218	0.205	0.288	0.171	0.180	0.157	0.250	0.198	0.253	0.224	0.186	0.172	0.162	0.000	
**Min29**	0.159	0.176	0.200	0.210	0.184	0.207	0.163	0.202	0.166	0.190	0.179	0.188	0.143	0.166	0.221	0.172	0.231	0.172	0.193	0.191	0.205	0.177	0.177	0.239	0.235	0.210	0.209	0.246	0.000

Table S5: Genetic diversity within hogweed Moscow invasive population obtained by combination of RAPD, ISSR and REMAP data

	Mosc1	Mosc2	Mosc3	Mosc4	Mosc5	Mosc6	Mosc7	Mosc8	Mosc9	Mosc10	Mosc11	Mosc12	Mosc13	Mosc14	Mosc15
Mosc1	0.000														
Mosc2	0.447	0.000													
Mosc3	0.531	0.378	0.000												
Mosc4	0.375	0.566	0.522	0.000											
Mosc5	0.333	0.404	0.385	0.333	0.000										
Mosc6	0.400	0.349	0.458	0.363	0.440	0.000									
Mosc7	0.393	0.469	0.370	0.440	0.457	0.500	0.000								
Mosc8	0.500	0.422	0.360	0.478	0.462	0.375	0.460	0.000							
Mosc9	0.360	0.349	0.333	0.364	0.400	0.391	0.569	0.292	0.000						
Mosc10	0.347	0.333	0.606	0.349	0.347	0.333	0.451	0.404	0.457	0.000					
Mosc11	0.255	0.273	0.429	0.244	0.333	0.362	0.358	0.551	0.519	0.348	0.000				
Mosc12	0.412	0.409	0.347	0.467	0.451	0.447	0.396	0.265	0.362	0.345	0.458	0.000			
Mosc13	0.422	0.526	0.442	0.436	0.467	0.366	0.404	0.442	0.366	0.550	0.381	0.333	0.000		
Mosc14	0.429	0.476	0.489	0.349	0.429	0.378	0.412	0.362	0.422	0.318	0.391	0.435	0.550	0.000	
Mosc15	0.250	0.388	0.222	0.360	0.321	0.385	0.217	0.333	0.346	0.333	0.321	0.358	0.404	0.333	0.000

Table S6: Genetic diversity within hogweed Peskovka invasive population obtained by combination of RAPD, ISSR and REMAP data

	Pesk1	Pesk2	Pesk3	Pesk4	Pesk5	Pesk6	Pesk7	Pesk8	Pesk9	Pesk10	Pesk11	Pesk12	Pesk13	Pesk14	Pesk15	Pesk16	Pesk17	Pesk18	Pesk19	Pesk20
Pesk1	0.000																			
Pesk2	0.469	0.000																		
Pesk3	0.393	0.257	0.000																	
Pesk4	0.451	0.385	0.474	0.000																
Pesk5	0.434	0.343	0.322	0.407	0.000															
Pesk6	0.600	0.438	0.464	0.490	0.678	0.000														
Pesk7	0.509	0.296	0.333	0.448	0.433	0.368	0.000													
Pesk8	0.519	0.382	0.400	0.418	0.333	0.481	0.410	0.000												
Pesk9	0.571	0.286	0.355	0.474	0.423	0.536	0.270	0.333	0.000											
Pesk10	0.551	0.460	0.418	0.480	0.577	0.224	0.393	0.472	0.527	0.000										
Pesk11	0.533	0.324	0.333	0.443	0.429	0.433	0.343	0.375	0.333	0.356	0.000									
Pesk12	0.448	0.333	0.406	0.424	0.410	0.483	0.292	0.355	0.313	0.474	0.265	0.000								
Pesk13	0.429	0.403	0.362	0.344	0.455	0.397	0.371	0.373	0.420	0.419	0.260	0.380	0.000							
Pesk14	0.410	0.333	0.284	0.419	0.313	0.574	0.382	0.446	0.403	0.600	0.437	0.333	0.324	0.000						
Pesk15	0.460	0.247	0.362	0.344	0.394	0.397	0.343	0.373	0.391	0.516	0.397	0.408	0.342	0.324	0.000					
Pesk16	0.370	0.441	0.467	0.491	0.368	0.630	0.508	0.379	0.500	0.585	0.438	0.355	0.493	0.385	0.373	0.000				
Pesk17	0.500	0.364	0.448	0.547	0.455	0.500	0.356	0.429	0.448	0.569	0.548	0.433	0.508	0.397	0.292	0.429	0.000			
Pesk18	0.451	0.342	0.294	0.429	0.385	0.484	0.362	0.394	0.500	0.541	0.417	0.371	0.333	0.260	0.253	0.364	0.344	0.000		
Pesk19	0.481	0.529	0.567	0.418	0.474	0.556	0.443	0.414	0.467	0.585	0.469	0.323	0.373	0.446	0.343	0.345	0.393	0.303	0.000	
Pesk20	0.538	0.455	0.448	0.434	0.491	0.462	0.390	0.500	0.448	0.608	0.419	0.433	0.354	0.365	0.323	0.536	0.407	0.375	0.357	0.000

Table S7: Genetic diversity within hogweed Kiev invasive population obtained by combination of RAPD, ISSR and REMAP data

	Kiev1	Kiev2	Kiev3	Kiev4	Kiev5	Kiev6	Kiev7	Kiev8	Kiev9	Kiev10	Kiev11	Kiev12	Kiev13	Kiev14	Kiev15	Kiev16	Kiev17
Kiev1	0.000																
Kiev2	0.404	0.000															
Kiev3	0.456	0.516	0.000														
Kiev4	0.485	0.483	0.347	0.000													
Kiev5	0.423	0.622	0.426	0.475	0.000												
Kiev6	0.429	0.441	0.373	0.360	0.417	0.000											
Kiev7	0.447	0.463	0.295	0.483	0.435	0.462	0.000										
Kiev8	0.416	0.505	0.400	0.444	0.459	0.429	0.485	0.000									
Kiev9	0.379	0.473	0.500	0.490	0.386	0.431	0.429	0.455	0.000								
Kiev10	0.396	0.381	0.333	0.407	0.468	0.432	0.600	0.379	0.484	0.000							
Kiev11	0.373	0.540	0.500	0.468	0.402	0.510	0.333	0.491	0.417	0.483	0.000						
Kiev12	0.500	0.545	0.546	0.495	0.429	0.495	0.477	0.346	0.443	0.511	0.484	0.000					
Kiev13	0.406	0.551	0.469	0.417	0.354	0.460	0.371	0.500	0.265	0.473	0.298	0.453	0.000				
Kiev14	0.420	0.364	0.505	0.432	0.490	0.475	0.455	0.346	0.443	0.444	0.570	0.447	0.516	0.000			
Kiev15	0.468	0.537	0.473	0.416	0.457	0.462	0.317	0.485	0.451	0.452	0.356	0.300	0.438	0.432	0.000		
Kiev16	0.462	0.435	0.406	0.455	0.451	0.437	0.478	0.333	0.465	0.404	0.505	0.286	0.434	0.408	0.522	0.000	
Kiev17	0.366	0.506	0.469	0.438	0.374	0.500	0.483	0.407	0.347	0.516	0.426	0.432	0.292	0.411	0.506	0.414	0.000

Table S8: Genetic diversity within hogweed Zhitomir industrial zone invasive population obtained by combination of RAPD, ISSR and REMAP data

	Zh(ind)1	Zh(ind)2	Zh(ind)3	Zh(ind)4	Zh(ind)5	Zh(ind)6	Zh(ind)7	Zh(ind)8	Zh(ind)9	Zh(ind)10	Zh(ind)11	Zh(ind)12	Zh(ind)13	Zh(ind)14
Zh(ind)1	0.000													
Zh(ind)2	0.344	0.000												
Zh(ind)3	0.469	0.487	0.000											
Zh(ind)4	0.397	0.388	0.624	0.000										
Zh(ind)5	0.407	0.467	0.447	0.420	0.000									
Zh(ind)6	0.397	0.405	0.419	0.333	0.375	0.000								
Zh(ind)7	0.365	0.387	0.491	0.410	0.388	0.640	0.000							
Zh(ind)8	0.385	0.469	0.389	0.396	0.449	0.424	0.242	0.000						
Zh(ind)9	0.424	0.418	0.416	0.421	0.533	0.471	0.514	0.406	0.000					
Zh(ind)10	0.424	0.538	0.216	0.453	0.449	0.531	0.407	0.467	0.453	0.000				
Zh(ind)11	0.519	0.579	0.590	0.520	0.600	0.536	0.513	0.379	0.456	0.455	0.000			
Zh(ind)12	0.328	0.275	0.504	0.353	0.318	0.414	0.317	0.457	0.492	0.364	0.276	0.000		
Zh(ind)13	0.496	0.446	0.565	0.480	0.574	0.528	0.540	0.415	0.429	0.529	0.431	0.581	0.000	
Zh(ind)14	0.469	0.504	0.431	0.452	0.512	0.452	0.456	0.420	0.487	0.450	0.504	0.520	0.304	0.000

Table S9: Genetic diversity within hogweed Zhitomir center invasive population obtained by combination of RAPD, ISSR and REMAP data

	Zh(c)1	Zh(c)2	Zh(c)3	Zh(c)4	Zh(c)5	Zh(c)6	Zh(c)7	Zh(c)8	Zh(c)9	Zh(c)10	Zh(c)11	Zh(c)12	Zh(c)13	Zh(c)14	Zh(c)15	Zh(c)16	Zh(c)17	Zh(c)18	Zh(c)19
Zh(c)1	0.000																		
Zh(c)2	0.483	0.000																	
Zh(c)3	0.560	0.406	0.000																
Zh(c)4	0.516	0.480	0.476	0.000															
Zh(c)5	0.443	0.440	0.610	0.520	0.000														
Zh(c)6	0.490	0.385	0.436	0.524	0.495	0.000													
Zh(c)7	0.458	0.500	0.462	0.475	0.466	0.472	0.000												
Zh(c)8	0.491	0.388	0.474	0.468	0.514	0.517	0.455	0.000											
Zh(c)9	0.538	0.424	0.393	0.477	0.358	0.474	0.482	0.475	0.000										
Zh(c)10	0.467	0.414	0.434	0.407	0.528	0.426	0.395	0.445	0.436	0.000									
Zh(c)11	0.451	0.429	0.475	0.468	0.417	0.465	0.474	0.505	0.456	0.481	0.000								
Zh(c)12	0.576	0.417	0.402	0.471	0.519	0.248	0.456	0.434	0.405	0.482	0.469	0.000							
Zh(c)13	0.375	0.419	0.462	0.495	0.406	0.472	0.440	0.491	0.407	0.450	0.495	0.534	0.000						
Zh(c)14	0.448	0.278	0.532	0.445	0.455	0.629	0.450	0.400	0.484	0.442	0.426	0.528	0.400	0.000					
Zh(c)15	0.485	0.488	0.352	0.540	0.412	0.477	0.446	0.532	0.468	0.382	0.500	0.481	0.606	0.421	0.000				
Zh(c)16	0.486	0.429	0.434	0.500	0.509	0.478	0.450	0.546	0.453	0.254	0.462	0.500	0.486	0.457	0.436	0.000			
Zh(c)17	0.464	0.392	0.486	0.520	0.333	0.439	0.465	0.477	0.450	0.473	0.355	0.442	0.465	0.471	0.490	0.436	0.000		
Zh(c)18	0.519	0.409	0.393	0.477	0.413	0.316	0.463	0.458	0.431	0.436	0.476	0.315	0.444	0.406	0.413	0.419	0.468	0.000	
Zh(c)19	0.479	0.419	0.558	0.475	0.505	0.434	0.480	0.491	0.519	0.486	0.432	0.495	0.260	0.400	0.505	0.505	0.505	0.444	0.000

Table S10: Genetic diversity within hogweed Caucasus native population obtained by combination of RAPD, ISSR and REMAP data

	Cau1	Cau2	Cau3	Cau4	Cau5	Cau6	Cau7	Cau8	Cau9	Cau10	Cau11	Cau12	Cau13	Cau14
Cau1	0000													
Cau2	0.548	0000												
Cau3	0.553	0.455	0000											
Cau4	0.566	0.405	0.528	0000										
Cau5	0.570	0.469	0.354	0.500	0000									
Cau6	0.580	0.651	0.494	0.526	0.581	0000								
Cau7	0.529	0.511	0.363	0.600	0.244	0.538	0000							
Cau8	0.506	0.578	0.516	0.425	0.511	0.564	0.585	0000						
Cau9	0.500	0.548	0.532	0.739	0.355	0.580	0.318	0.529	0000					
Cau10	0.535	0.538	0.500	0.506	0.473	0.620	0.566	0.325	0.458	0000				
Cau11	0.500	0.567	0.492	0.494	0.464	0.553	0.467	0.461	0.413	0.511	0000			
Cau12	0.485	0.392	0.515	0.565	0.529	0.511	0.468	0.532	0.546	0.516	0.426	0000		
Cau13	0.515	0.370	0.505	0.531	0.481	0.479	0.520	0.540	0.515	0.564	0.402	0.339	0000	
Cau14	0.511	0.495	0.520	0.596	0.556	0.586	0.538	0.560	0.553	0.522	0.490	0.379	0.358	0000

1. Osipova, E.S.; Koveza, O.V.; Troitskij, A.V.; Dolgikh, Y.I.; Shamina, Z.B.; Gostimskij, S.A. Analysis of specific RAPD and ISSR fragments in maize (Zea mays L.) somaclones and development of SCAR markers on their basis. *Russ. J. Genet.* **2003**, *39*, 1412–1419, doi:10.1023/B:RUGE.0000009156.74246.bc.
2. Motawei, M.I.; Al–Doss, A.A.; Moustafa, K.A. Genetic diversity among selected wheat lines differing in heat tolerance using molecular markers. *J. Food, Agric. Environ*. **2007**, *5*, 180–183.
3. El Maati F.B.; Jlibéne, M.; Moumni M. Study of the polymorphism of common wheat using ISSR markers. *Int. J. food, Agric. Environ.* **2004**, *2*, 121–125.
4. Dolgikh, Yu.I.; Solov'eva, A.I.; Tereshonok, D.V. Variability of DNA markers in Arabidopsis thaliana cultured cells under standard growing conditions and under the influence of stressors. *Biology Bulletin* **2017**, *44*, 120–127, doi:10.1134/S1062359017020042.
5. Galaev, A.V.; Babayants, L.T.; Sivolap, Y.M. Detection of the introgression of genome elements of the Aegilops cylindrica Host. into the Triticum aestivum L. genome by ISSR and SSR analysis. *Genetika* **2004**, *40*, 1654–1661, doi:10.1007/s11177-005-0007-y.
6. Queen, R.A.; Gribbon, B.M.; James, C.; Jack, P.; Flavell, A.J. Retrotransposon-based molecular markers for linkage and genetic diversity analysis in wheat. *Mol. Genet. Genomics* **2004**, *271*, 91–97, doi:10.1007/s00438-003-0960-x.
